# Assessment of Dental Age of Children Aged 3.5 to 16.9 Years Using Demirjian’s Method: A Meta-Analysis Based on 26 Studies

**DOI:** 10.1371/journal.pone.0084672

**Published:** 2013-12-18

**Authors:** Jin Yan, Xintian Lou, Liming Xie, Dedong Yu, Guofang Shen, Yilin Wang

**Affiliations:** 1 Department of Stomatology, Punan Hospital of Pudong New District, Shanghai, China; 2 Department of Oral and Craniomaxillofacial Science, Ninth People’s Hospital, College of Stomatology, Shanghai Jiao Tong University School of Medicine, Shanghai Key Laboratory of Stomatology, Shanghai, China; University of Toronto, Canada

## Abstract

**Background** A method for assessing dental maturity in different populations was first developed in 1973 by Demirjian and has been widely used and accepted since then. While the accuracy for evaluating dental age using Demirjian’s method compared to children’s chronological age has been extensively studied in recent years, the results currently available remain controversial and ambiguous.

**Methods** A literature search of PubMed, Embase, Web of Science, CNKI and CBM databases was conducted to identify all eligible studies published before July 12th, 2013. Weighted mean difference (WMD) with corresponding 95% confidence interval (95% CI) was used to evaluate the applicability of Demirjian’s method for estimating chronological age in children.

**Results:** A meta-analysis was conducted on 26 studies with a total of 11,499 children (5,301 boys and 6,198 girls) aged 3.5 to 16.9 years. Overall, we found that Demirjian’s method overestimated dental age by 0.35 (4.2 months) and 0.39 (4.68 months) years in males and females, respectively. A subgroup analysis by age revealed that boys and girls between the ages of 5 to 14 were given a dental age estimate that was significantly more advanced than their chronological age. Differences between underestimated dental ages and actual chronological ages were lower for male and female 15- and 16-year-old subgroups, though a significant difference was found in the 16-year-old subgroup.

**Conclusions** Demirjian’s method’s overestimation of actual chronological tooth age reveals the need for population-specific standards to better estimate the rate of human dental maturation.

## Introduction

Age determination is of particular interest in orthodontics and paediatric dentistry for making accurate diagnoses and treatment strategies [1]. It is generally accepted that several indicators of somatic development, including skeletal, dental and menarche ages, somatic maturity, sexual maturation, body height and weight, can be used to determine the chronological age and to assess the growth and development of children [2]. However, dental maturity indicators have received more attention and are thought to be more useful indices of maturation since they exhibit less variability than other bone and skeletal tissues, which are more susceptible to exogenic factors, such as malnutrition or systematic diseases [3,4].

The two major approaches used to estimate dental age are the stage of tooth eruption in the oral cavity and the pattern of tooth development observed in radiographs [5,6]. Measuring the stage of dental eruption is not a currently preferred method because tooth eruption is a discontinuous process, in contrast to tooth calcification, which is an ongoing process [7]. Moreover, this method cannot be used with children who have not yet reached the stage of mixed dentition. In addition, this method is affected by various local factors, such as crowding, extractions, ankylosis, ectopic positions, and persistence of primary teeth [8]. Stages of tooth formation, however, are less affected by local factors and can be assessed using radiographs on a broader age range of children [9]. Thus, tooth formation, for the reasons mentioned above, should be considered as a more reliable criterion for determining dental maturation than tooth eruption.

Various methods employed for determining dental age are based on the degree of the calcification observed in radiographic examinations of permanent teeth [10-14]. Among these proposed methods, one of the most widely applied methods for ascertaining dental age is the eight stage system introduced by Demirjian et al. (subsequently referred as Demirjian’s method) [12]. With this system, the development of seven left mandibular permanent teeth (except the third molar) are observed using panoramic radiographs and classified by means of an eight-stage system (ranging from A to H; and an additional stage 0, which represents no signs of calcification) (Demirjian, 1978). Each stage of each of the seven teeth is assigned a numeric value, which differs according to the sex of the individual based on the conversion table provided by Demirjian. Summing the obtained scores of the seven teeth produces a total maturity score, which is then converted into an estimated dental age using the conversion table.

Demirjian’s method, however, was formulated using 21,328 French-Canadian children between 2 and 20 years of age. Being the most widely used dental age examination method, numerous studies have tested the applicability of this method in various populations, including Chinese [15-17], Turkish [18-20], Caucasian American [21], South Indian [22-24], Belgian [25], Dutch [7], Brazilian [26,27], Australian [28], and British [17,29,30]. A considerable number of studies have reported that Demirjian’s method overestimated age in their respective populations. Mani et al.’s study on 6.5- to 15.5-year-old children from Malaysia found that using Demirjian’s method resulted in overestimations of 0.75 and 0.61 years for boys and girls, respectively [4]. Jayaraman et al. recently tested the method with Southern Chinese children between the ages of 3-16 and found a mean overestimation of 0.62 years for boys and 0.36 years for girls [17]. On the other hand, a few studies suggest that Demirjian’s method achieves accurate estimations for populations besides French-Canadian [25,29,30]. Hegde et al. employed Demirjian’s method for estimating dental age in 6- to 12.9-year-old Belgian children and reported that this method only overestimated ages by 0.14 year (51 days) for boys and 0.04 year (15 days) for girls and thus concluded that this method was applicable in Belgian children [25].

While a large number of studies on Demirjian’s method have been published, to the best of our knowledge, no meta-analysis has been conducted to evaluate the overall accuracy of Demirjian’s system for predicting the age of children. Thus, we conducted this systematic review in order to better understand the accuracy of Demirjian’s method to evaluate dental age. By comparing dental age estimates against the standard of chronological age, our study contributes to a better understanding of the relationship between estimated dental age and chronological age and how this relationship is modulated by gender, ethnicity and age.

## Materials and Methods

To ensure scientific rigor, the current meta-analysis was designed according to the Preferred Reporting Items for Systematic Reviews and Meta-analyses (PRISMA) guidelines (Table S1).

### Search Strategy

A literature search of relevant studies published up to July 12th, 2013 was conducted in PubMed, Embase, Web of Science, CNKI (Chinese National Knowledge Infrastructure), and CBM (Chinese Biomedical Literature Database) databases. No limit on publication language was used. The following combined search terms were used: (‘tooth’ or ‘teeth’ or ‘age determination by teeth’) and (‘radiography, panoramic’ or ‘pantomography’ or ‘panoramic radiography’) and (‘child’ or ‘child, preschool’ or ‘adolescent’). We also screened the reference lists of retrieved articles to identify additional potential sources.

### Inclusion and Exclusion Criteria

Studies had to meet the following three criteria to be included in this meta-analysis: (i) a cross-sectional or retrospective study evaluating the accuracy and precision of dental age using Demirjian’s method to estimate chronological age; (ii) all subjects were healthy, without any developmental disorders and retained all the mandibular permanent teeth (erupted or un-erupted); (iii) inclusion of sufficient data on the size of the sample and mean (SD) values of dental and chronological ages.

The following exclusion criteria were also applied to potential studies: (i) duplicate publications, case reports, letters, reviews or editorial articles; (ii) included subjects with growth disorders or chronic diseases, or lacked information on subjects’ health status; (iii) absence of accurate and reproducible parameters, such as dental and chronological age differences. Additionally, when the data was included in multiple studies using the same case series, either the study with the most recent publication or the largest sample size was considered. Studies were reviewed independently by two authors and disagreements were resolved through discussion.

### Data extraction and Quality Assessment

For studies that fulfilled the inclusion criteria, two reviewers independently extracted and entered data into a structured data table. The following data were recorded: the first author’s surname, year of publication, country of origin, ethnic subgroups, research type, number of subjects, sex ratio of the subjects, age range, method used for dental age assessment, dental age and chronological age (mean and SD). In addition, we compared key study characteristics to determine the existence of multiple publications from the same study. In addition, all eligible articles were read and scored for quality by two independent researchers using the modified STROBE quality score system [31]. The criteria employed are shown in Table S2. Forty assessment items matching quality appraisals were used in this meta-analysis, with scores ranging from 0 to 40. Studies with at least 70% of the total quality score (28 out of 40) were considered high-quality.

### Statistical Analysis

We evaluated the applicability of Demirjian’s method for estimating chronological ages of boys and girls independently since Demirjian’s method provides separate standards to account for sexual differences (Table S3). The weighted mean difference (WMD) with a corresponding 95% confidence interval (95% CI) was used to calculate and assess the accuracy of Demirjian’s method’s dental age estimates compared with children’s actual chronological ages. Between-study variation and heterogeneity were estimated using Cochran’s *Q*-statistic, with *P* < 0.05 indicating statistically significant heterogeneity [32]. We also quantified the effect of heterogeneity using the *I* ^2^ test (ranges from 0 to 100%), which determines the proportion of inter-study variability that can be attributed to heterogeneity rather than chance [33]. The random effects model (*DerSimonian-Laird* method) was used, except when a significant *Q*-test (*P* > 0.05) or *I* ^2^ < 50% indicated the absence of heterogeneity among studies, in which case the fixed effects model (*Mantel-Haenszel* method) was applied. Inter-study variation was explored by subgrouping data according to gender (i.e., female or male), ethnicity (i.e., Caucasian or Asian), and age (i.e., from 5 up to and including 16 years of age), if feasible. In our statistical analysis, the age group of 3- to 4-year-olds for both boys and girls were excluded due to its small sample size.

In order to ensure the reliability of results, a sensibility analysis was performed to evaluate influences. Publication bias was visually evaluated using a Begg’s funnel plot, in which effect estimates of the common outcome measure were plotted against trial sample size. In addition, Egger’s linear regression test, which measures funnel plot asymmetry via a natural logarithm scale of WMD, was used to evaluate publication bias [34]. All results were reported with 95% CI and all *P*-values were two-sided. Analyses were conducted with the STATA Version 12.0 software (Stata Corp, College Station, TX).

## Results

### The Characteristics of Included Studies

**Figure 1:**
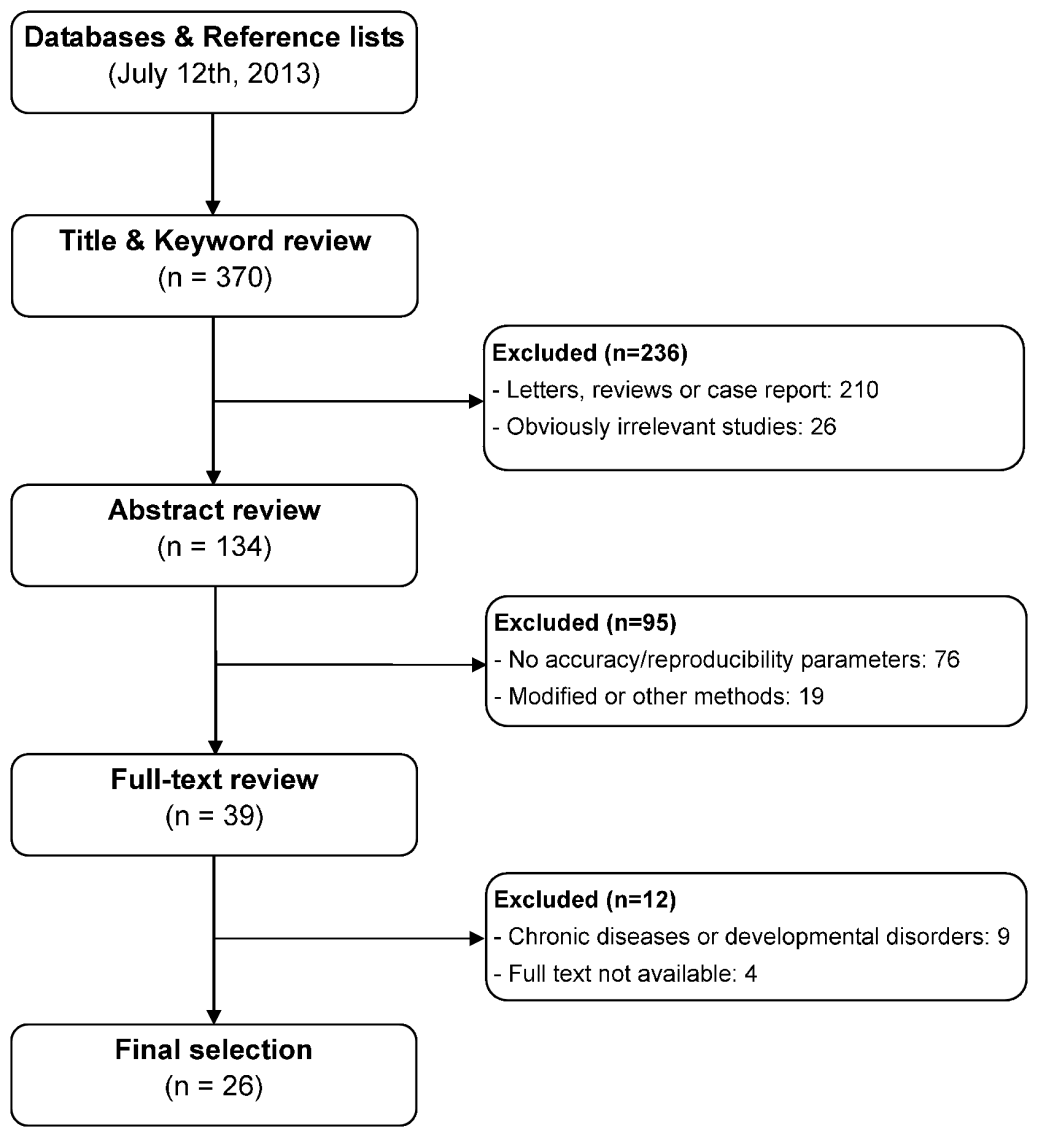
Flow diagram of the selection process of the included studies and the specific reasons for exclusion from the present meta-analysis.

A total of 370 peer-reviewed articles were retrieved through our database search. In accordance with our criteria, 26 studies [4,7,15-30,35-42] were included in the present meta-analysis and 344 were excluded. Figure 1 displays a flow chart of the study selection process, as well as the specific reasons for exclusion according to the PRISMA statement. Of the 26 included studies, 19 were cross-sectional studies, 5 were retrospective studies, and the other 2 were retrospective cross-sectional studies. Of the eligible studies, there were 15 studies with a total of 6,333 Caucasians children (2,956 boys and 3,377 girls), and 11 studies with a total of 5,166 Asians children (2,345 boys and 2,821 girls). The qualities of all included studies were moderately high, with STROBE scores greater than 20. The characteristics of included studies are summarized in Table 1. Our findings on the accuracy of Demirjian’s method’s dental age evaluation are reported below according to gender.

**Table 1:** Characteristics of the studies that examine the accuracy of dental age compared with chronological age in children.

**First author**	**Year**	**Location**	**Ethnicity**	**Research type**	**No. of subjects**	**Male (%)**	**Age range (yrs)**	**Method**	**STORBE score**
Koshy S	1998	India	Asian	Cross-sectional	184	50.0%	5-14.9 y.o.	Demirjian's 1973	23
Hegde RJ	2002	Belgium	Caucasian	Cross-sectional	197	47.7%	6-12.9 y.o.	Demirjian's 1976	25
Eid RM	2002	Brazil	Caucasian	Retrospective study	689	46.6%	6-14.9 y.o.	Demirjian's 1973	27
Leurs IH	2005	Dutch	Caucasian	Retrospective study	451	50.1%	3.5-17 y.o.	Demirjian's 1978	26
Tao J	2007	China	Asian	Cross-sectional	633	33.7%	11-16.9 y.o.	Demirjian's 1973	22
Mani SA	2008	Malaysia	Asian	Cross-sectional	428	50.0%	6.7-15.5 y.o.	Demirjian's 1973	28
Al-Emran S	2008	Saudi	Asian	Retrospective study	490	45.9%	8.5-15.5 y.o.	Demirjian's 1973	27
Uysal TA	2009	Turkey	Caucasian	Cross-sectional	50	50.0%	8-13 y.o.	Demirjian's 1978	26
Shi GF	2009	China	Asian	Cross-sectional	473	34.5%	11-16.9 y.o.	Demirjian's 1973	23
Mitchell JC	2009	UK	Caucasian	Cross-sectional	50	44.0%	15-17 y.o.	Demirjian's 1973	25
Maia MC	2010	Brazil	Caucasian	Retrospective cross-sectional	1491	44.9%	7-13.9 y.o.	Demirjian's 1973	28
Bagherpour A	2010	Iran	Asian	Cross-sectional	311	45.3%	6-12.9 y.o.	Demirjian's 1973	27
Bagherian A	2011	Iran	Asian	Cross-sectional	519	50.9%	3.5-13.5 y.o.	Demirjian's 1973	26
Celikoglu MK	2011	Turkey	Caucasian	Cross-sectional	807	45.4%	7-14.9 y.o.	Demirjian's 1973	28
Flood SJ	2011	Australia	Caucasian	Cross-sectional	143	58.0%	4.5-14.5 y.o.	Demirjian's 1973	29
Jayaraman JN	2011	China	Asian	Cross-sectional	182	50.0%	3-15.9 y.o.	Demirjian's 1973	25
Nik-Hussein NN	2011	Malaysia	Asian	Retrospective cross-sectional	991	50.9%	4.5-15.5 y.o.	Demirjian's 1973	23
Rozylo IA	2011	Poland	Caucasian	Cross-sectional	718	40.0%	6-17 y.o.	Demirjian's 1973	24
Weddell LS	2011	USA	Caucasian	Cross-sectional	257	45.5%	5.5-17.5 y.o.	Demirjian's 1976	26
Yadava MG	2011	UK	Caucasian	Cross-sectional	100	50.0%	9-11 y.o.	Demirjian's 1978	27
Sukhia RH	2012	Pakistan	Asian	Cross-sectional	822	51.9%	7-14.9 y.o.	Demirjian's 1973	26
Malik PR	2012	India	Asian	Cross-sectional	100	0.0%	8-14 y.o.	Demirjian's 1973	28
Kirzioglu Z	2012	Turkey	Caucasian	Retrospective study	425	49.9%	7-13 y.o.	Demirjian's 1973	29
Nur BA	2012	Turkey	Caucasian	Retrospective study	673	50.8%	5-15.9 y.o.	Demirjian's 1976	26
Grover SC	2012	India	Asian	Cross-sectional	215	47.4%	6-15 y.o.	Demirjian's 1973	25

### Comparisons between Dental Age and Chronological Age in Males

**Figure 2:**
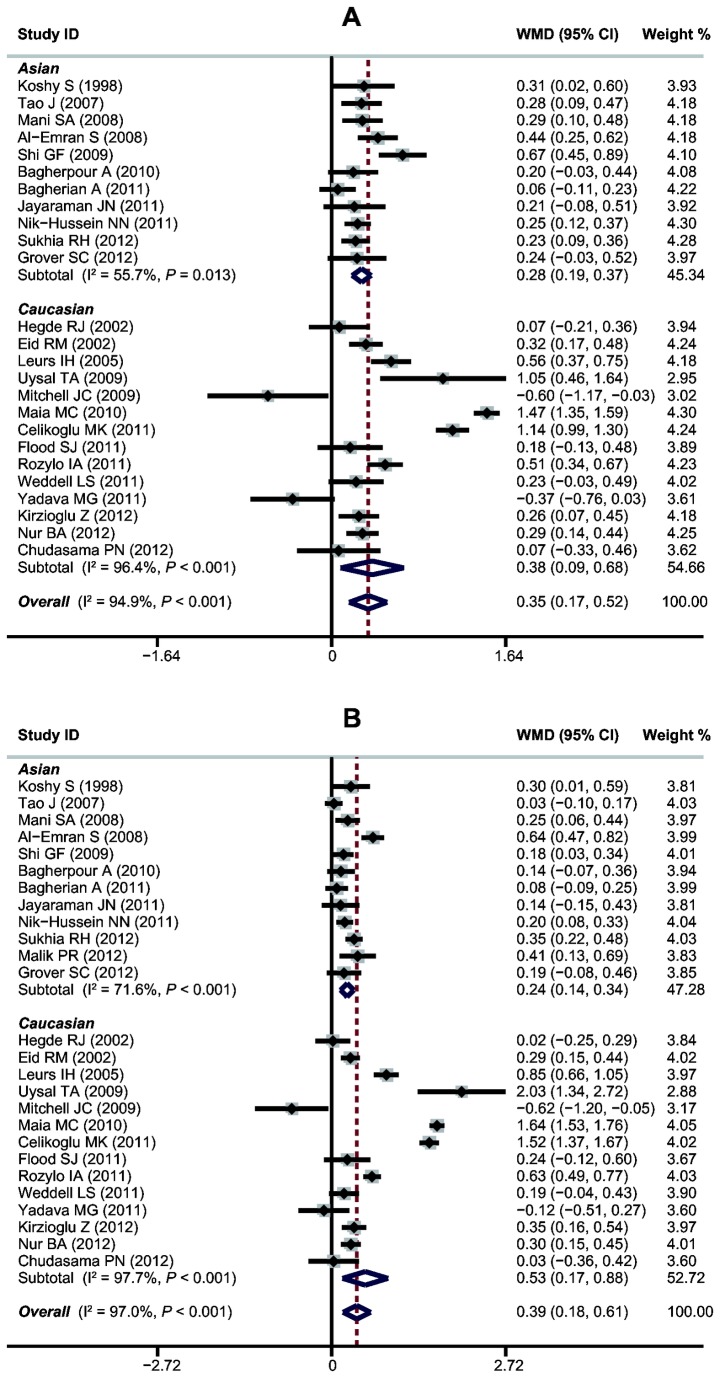
Forest plot of WMDs for the comparison between dental age using Demirjian’s method and chronological age among boys (A) and girls (B).

A summary of the meta-analysis findings on the inter-relationship between estimated dental age using Demirjian’s method and the chronological age for males is presented in Table 2. Since heterogeneity obviously existed, the random effects model was used. Overall, the initial meta-analysis showed a significant difference between the dental age and the chronological age for males (WMD = 0.35, 95%CI = 0.17-0.52, *P* < 0.001). When stratified analysis by ethnicity was performed, the mean difference between the dental age determined from the French-Canadian standards and the chronological age was 0.28 years for Asians (95%CI = 0.19-0.37, *P* < 0.001) and 0.38 years for Caucasians (95%CI = 0.09-0.68, *P* = 0.011) (Figure 2A). Further analysis by age groups suggests that, in most subgroups, there were statistically significant differences between the chronological age and the dental age. As shown in Table 2, statistically significant overestimation was noted in the first ten age subgroups (i.e., from 5 to 14 years of age), and underestimation of age was uncommon and seen only in the other two subgroups (i.e., in the 15- and 16-year-old subgroups). The underestimation, however, was significant only in age group 16- (WMD= -1.66, 95%CI = -2.21, -1.11, *P* < 0.001). Only age group 15- in boys showed no statistically significant differences between the chronological age and the dental age.

**Table 2:** Meta-analysis comparing the between dental age using Demirjian’s method and chronological age (in years) among boys.

**Subgroups**	**No. of study**	**No. of subjects**	**WMD**	**95%CI**	***P* value**	***P_h_***	**Power**
***Ethnicity***							
Asian	10	2,352	0.28	(0.19, 0.37)	<0.001	0.013	1.000
Caucasian	15	2,956	0.38	(0.09, 0.68)	0.011	<0.001	1.000
***Age groups***							
5-5.9 y.o.	3	31	1.97	(1.28, 2.65)	<0.001	<0.001	0.153
6-6.9 y.o.	5	76	1.38	(1.01, 1.76)	<0.001	<0.001	0.586
7-7.9 y.o.	8	211	1.42	(1.20, 1.64)	<0.001	0.022	0.946
8-8.9 y.o.	8	292	0.77	(0.60, 0.94)	<0.001	<0.001	1.000
9-9.9 y.o.	8	252	0.85	(0.66, 1.04)	<0.001	<0.001	1.000
10-10.9 y.o.	8	349	0.93	(0.77, 1.09)	<0.001	<0.001	1.000
11-11.9 y.o.	10	334	0.92	(0.76, 1.09)	<0.001	<0.001	1.000
12-12.9 y.o.	10	420	0.94	(0.80, 1.09)	<0.001	<0.001	1.000
13-13.9 y.o.	8	334	0.78	(0.61, 0.94)	<0.001	<0.001	1.000
14-14.9 y.o.	7	180	0.85	(0.62, 1.07)	<0.001	0.002	1.000
15-15.9 y.o.	4	78	0.00	(-0.33, 0.32)	0.978	0.456	1.000
16-16.9 y.o.	2	35	-1.66	(-2.21, -1.11)	<0.001	<0.001	0.220
***Overall***	25	5,308	0.35	(0.17, 0.52)	<0.001	<0.001	1.000

### Comparisons between Dental Age and Chronological Age in Females

Table 3 compares the estimated chronological ages for females overall and at each age group using Demirjian’s method. Since heterogeneity was obvious (all *P* < 0.05), the random effects model was used for combining study estimates. The overall mean difference between estimated dental age and chronological age for girls was 0.39 (95%CI: 0.18-0.61, *P* < 0.001) years. Subgroup analyses by ethnicity showed that the mean difference between the chronological age and dental age is 0.24 years for Asians and 0.52 years for Caucasians (Figure 2B). An analysis of the difference between dental age and chronological age for girls in different age groups was also conducted. Except for the 15- and 16-year-old subgroups, in which age was underestimated, dental age was overestimated in all age groups by between 0.01 and 1.63 years. The largest overestimations were made for 6- and 13-year-old subgroups, which had overestimates ranging between 0.51 and 2.11 years. Among all the age subgroups, the least difference in dental age was observed in 15 year-old girls.

**Table 3:** Meta-analysis comparing the between dental age using Demirjian’s method and chronological age (in years) among girls.

**Subgroups**	**No. of study**	**No. of subjects**	**WMD**	**95%CI**	***P* value**	***P_h_***	**Power**
***Ethnicity***							
Asian	11	2,871	0.24	(0.14, 0.34)	<0.001	<0.001	1.000
Caucasian	15	3,374	0.52	(0.17, 0.88)	0.004	<0.001	1.000
***Age groups***							
5-5.9 y.o.	3	28	1.11	(0.01, 2.21)	0.047	0.048	0.425
6-6.9 y.o.	5	62	1.31	(0.74, 1.63)	<0.001	<0.001	0.634
7-7.9 y.o.	8	238	0.91	(0.52, 1.31)	<0.001	0.001	1.000
8-8.9 y.o.	8	296	1.05	(0.63, 1.47)	<0.001	<0.001	1.000
9-9.9 y.o.	8	281	0.87	(0.50, 1.24)	<0.001	<0.001	1.000
10-10.9 y.o.	8	404	1.00	(0.70, 1.31)	<0.001	0.003	1.000
11-11.9 y.o.	10	442	1.07	(0.82, 1.32)	<0.001	<0.001	1.000
12-12.9 y.o.	10	568	0.86	(0.40, 1.31)	<0.001	0.006	1.000
13-13.9 y.o.	8	492	1.31	(0.51, 2.11)	0.001	<0.001	1.000
14-14.9 y.o.	7	243	0.78	(0.06, 1.49)	0.032	<0.001	1.000
15-15.9 y.o.	4	98	-0.58	(-1.17, -0.01)	0.051	0.017	1.000
16-16.9 y.o.	2	61	-0.87	(-1.23, -0.50)	0.001	0.005	0.931
***Overall***	26	6,245	0.39	(0.18, 0.61)	<0.001	<0.001	1.000

### Evaluation of Heterogeneity and Publication Bias

**Figure 3:**
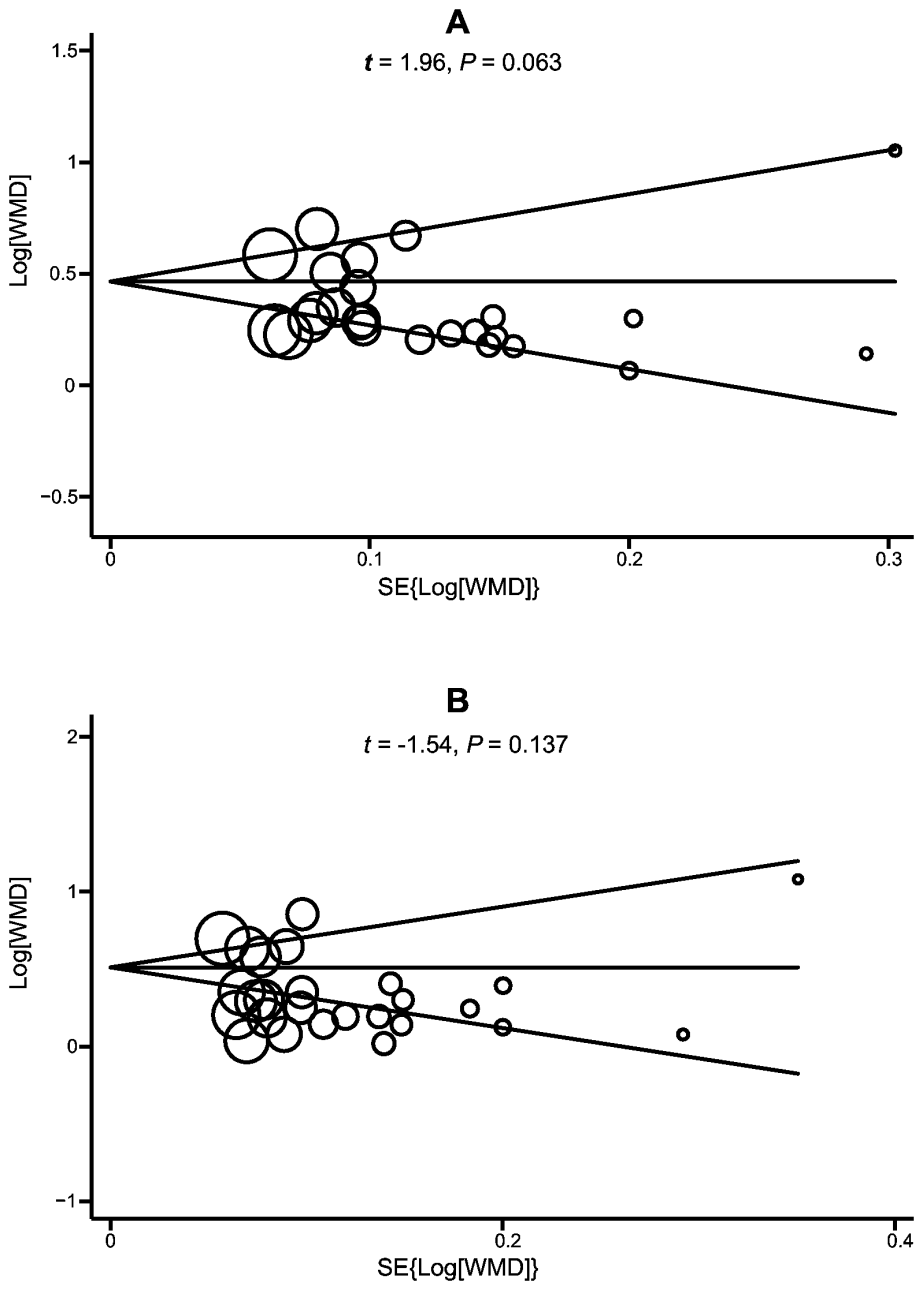
Begg’s funnel plot of the meta-analysis of the difference between dental age using Demirjian’s method and chronological age among boys (A) and girls (B). Each point represents a separate study for the indicated association. Log[WMD], natural logarithm of WMD. SE{Log[WMD]}, standard error of natural logarithm of WMD. Horizontal line, mean magnitude of the effect.

A sensitivity analysis was also performed to assess the influence of each individual study on the pooled WMD by omitting each individual studies in turn. No statistically significant differences were observed when comparing these results with the overall analysis. Funnel plots and Egger’s linear regression test were used to assess potential publication bias of the included studies. The shapes of the funnel plots did not reveal any evidence of obvious asymmetry (Figure 3). Egger’s test also did not display strong statistical evidence of publication bias (boys: *t* = 1.96, *P* = 0.063; girls: *t* = -1.54, *P* = 0.137).

## Discussion

Methods for determining a child’s growth and development are of great value for both forensic odontology and anthropologic purposes. Tooth development, which was only slightly affected by systemic factors such as endocrine and nutritional status, corresponds well with the chronological age [4]. Thus, understanding the tooth development stages and the associated detail has long been used for determination of chronological age. One of the most well-known and commonly used methods for ascertaining dental age is Demirjian’s eight-stage method [12], which was originally applied to a large sample of Canadian children in 1973. In recent years, the applicability of this method has been extensively investigated in various populations, raising doubts about the credibility of this method. Therefore, we conducted a meta-analysis to explore this issue. The main objective of this study is to determine the mean deviations between estimated and actual chronological ages (at each age group and overall) of children when applying Demirjian’s method.

A total of 11,499 children (5,301 boys and 6,198 girls) from 26 published studies were included in this meta-analysis. Our findings show that Demirjian’s method tends to estimate dental age as more advanced than chronological age. The overestimation of dental age varied between 0.78 to 1.97 years for boys (mean 0.35 years), and 0.86 to 1.31 years for girls (mean 0.39 years), indicating that the females may show earlier maturation in dental development than the males. Considering mean differences between estimated dental ages and chronological ages, most authors have also found statistically significant differences of 0.681 years and 0.616 years [26], 0.4 years and 0.6 years [7], 0.3 years and 0.4 years [35], 0.75 years and 0.61 years [4], 0.34 years and 0.25 years [36], 0.66 years and 0.56 years [23] in boys and girls, respectively. Moreover, when a stratified analysis based on ethnicity was conducted, an average overestimation of 0.28 years and 0.38 years were found in Asian and Caucasian boys, and 0.24 years and 0.52 years were found in Asian and Caucasian girls, respectively. Results from our meta-analysis suggest that while tooth development may be used to estimate age, variations between populations, which are sensitive to gender, must be accounted for. This advancement in dental maturation may partly be explained by positive trends in growth and development observed in the past decades since the standards were established in the French-Canadian population [43]. Furthermore, although dental development is thought to be less affected by extrinsic or environmental factors, such as nutrition, it is known to be more affected by genetic and ecological factors than other growth measures.

From the subgroup analysis of differences in dental age and chronological age separated by age and gender, dental age was shown to be overestimated in the majority of subgroups. As shown in Tables 2 and 3, a greater overestimation was observed in the younger age groups from ages 5 through 14 for both genders. This may be account for difficulties in predicting the growth of younger children. However, boys and girls at 15 and 16 years-old displayed a delayed dental age compared to French-Canadian children. This may be due to the unsteady and non-uniform process of dental growth that is associated with the pre-pubertal or pubertal growth changes during this age period [4]. Alternatively, because of the small sample sizes of 15 and 16 year-old children included in the analysis, this result may lack statistical power.

In interpreting the results of this meta-analysis, some specific issues should be addressed. First, the sample sizes of age subgroups were relatively modest, so the statistical power of the association analysis was inevitably low. Additionally, most of the included studies were carried out in Asian and Caucasian populations; thus, research on other populations needs to be conducted. Due to the shortcomings of the present study, our findings demand testing and verification by further studies. Aside from the limitations listed above, our meta-analysis has some strength. To the best of our knowledge, this is the first meta-analysis on this topic. Further, this study explores inter-study variations through subgroup analyses according to ethnicity, gender, and age.

In conclusion, Demirjian’s method overestimates dental age in almost every age group for children of both genders between 5 to 14 years old. In addition, ethnic differences were also found to affect the accuracy of Demirjian’s method. Therefore it is important to develop different estimation calculations based on local population characteristics in order to obtain accurate estimations of dental age.

## Supporting Information

Table S1 **PRISMA Checklist for systematic review and meta-analysis.**
(DOC)

Table S2 **The modified STROBE quality score systems.**
(DOC)

Table S3 **The different versions of Demirjian’s method (1973 and 1976).**
(DOC)
