# Supplementary material for: Assessment of Dental Age of Children Aged 3.5 to 16.9 Years Using Demirjian’s Method: A Meta-Analysis Based on 26 Studies
Source: PLoS One. 2013 Dec 18;8(12):e84672. doi: 10.1371/journal.pone.0084672 (PMC3867507; doi:10.1371/journal.pone.0084672)
Supplement: Table S3 — The different versions of Demirjian’s method (1973 and 1976). (DOC) [file pone.0084672.s003.doc]

**Supplement Table S3**

**Part1**. Scores for dental stages 7 teeth (mandibular left side) in Demirjian’s 1973 method

| **Boys** | | | | | | | | | |
| --- | --- | --- | --- | --- | --- | --- | --- | --- | --- |
|  | **Stage** | | | | | | | | |
| **Tooth** | 0 | A | B | C | D | E | F | G | H |
| **M_2_** | 0.0 | 2.1 | 3.5 | 5.9 | 10.1 | 12.5 | 13.2 | 13.6 | 15.4 |
| **M_1_** |  |  |  | 0.0 | 8.0 | 9.6 | 12.3 | 17.0 | 19.3 |
| **PM_2_** | 0.0 | 1.7 | 3.1 | 5.4 | 9.7 | 12.0 | 12.8 | 13.2 | 14.4 |
| **PM_1_** |  |  | 0.0 | 3.4 | 7.0 | 11.0 | 12.3 | 12.7 | 13.5 |
| **C** |  |  |  | 0.0 | 3.5 | 7.9 | 10.0 | 11.0 | 11.9 |
| **I_2_** |  |  |  | 0.0 | 3.2 | 5.2 | 7.8 | 11.7 | 13.7 |
| **I_1_** |  |  |  |  | 0.0 | 1.9 | 4.1 | 8.2 | 11.8 |
| **Girls** | | | | | | | | | |
|  | **Stage** |  |  |  |  |  |  |  |  |
| **Tooth** | 0 | A | B | C | D | E | F | G | H |
| **M_2_** | 0.0 | 2.7 | 3.9 | 6.9 | 11.1 | 13.5 | 14.2 | 14.5 | 15.6 |
| **M_1_** |  |  |  | 0.0 | 4.5 | 6.2 | 9.0 | 14.0 | 16.2 |
| **PM_2_** | 0.0 | 1.8 | 3.4 | 6.5 | 10.6 | 12.7 | 13.5 | 13.8 | 14.6 |
| **PM_1_** |  |  | 0.0 | 3.7 | 7.5 | 11.8 | 13.1 | 13.4 | 14.1 |
| **C** |  |  |  | 0.0 | 3.8 | 7.3 | 10.3 | 11.6 | 12.4 |
| **I_2_** |  |  |  | 0.0 | 3.2 | 5.6 | 8.0 | 12.2 | 14.2 |
| **I_1_** |  |  |  |  | 0.0 | 2.4 | 5.1 | 9.3 | 12.9 |

**Source:** Demirjian A, Goldstein H, Tanner JM (1973) A new system of dental age assessment. Hum Biol 45: 211-227.

**Part 2**. Scores for dental stages of 7 teeth (mandibular left side) in Demirjian’s 1976 method

| **Boys** | | | | | | | | | |
| --- | --- | --- | --- | --- | --- | --- | --- | --- | --- |
|  | **Stage** | | | | | | | | |
| **Tooth** | 0 | A | B | C | D | E | F | G | H |
| **M_2_** | 0.0 | 1.7 | 3.1 | 5.4 | 8.6 | 11.4 | 12.4 | 12.8 | 13.6 |
| **M_1_** |  |  |  | 0.0 | 5.3 | 7.5 | 10.3 | 13.9 | 16.8 |
| **PM_2_** | 0.0 | 1.5 | 2.7 | 5.2 | 8.0 | 10.8 | 12.0 | 12.5 | 13.2 |
| **PM_1_** |  | 0.0 | 4.0 | 6.3 | 9.4 | 13.2 | 14.9 | 15.5 | 16.1 |
| **C** |  |  |  | 0.0 | 4.0 | 7.8 | 10.1 | 11.4 | 12.0 |
| **I_2_** |  |  |  | 0.0 | 2.8 | 5.4 | 7.7 | 10.5 | 13.2 |
| **I_1_** |  |  |  | 0.0 | 4.3 | 6.3 | 8.2 | 11.2 | 15.1 |
| **Girls** | | | | | | | | | |
|  | **Stage** |  |  |  |  |  |  |  |  |
| **Tooth** | 0 | A | B | C | D | E | F | G | H |
| **M_2_** | 0.0 | 1.8 | 3.1 | 5.4 | 9.0 | 11.7 | 12.8 | 13.2 | 13.8 |
| **M_1_** |  |  |  | 0.0 | 3.5 | 5.6 | 8.4 | 12.5 | 15.4 |
| **PM_2_** | 0.0 | 1.7 | 2.9 | 5.4 | 8.6 | 11.1 | 12.3 | 12.8 | 13.3 |
| **PM_1_** |  | 0.0 | 3.1 | 5.2 | 8.8 | 12.6 | 14.3 | 14.9 | 15.5 |
| **C** |  |  |  | 0.0 | 3.7 | 7.3 | 10.0 | 11.8 | 12.5 |
| **I_2_** |  |  |  | 0.0 | 2.8 | 5.3 | 8.1 | 11.2 | 13.8 |
| **I_1_** |  |  |  | 0.0 | 4.4 | 6.3 | 8.5 | 12.0 | 15.8 |

**Source:** Demirjian A, Goldstein H (1976) New systems for dental maturity based on seven and four teeth. Ann Hum Biol 3: 411-421.

**Part 3**. Scores for dental stages of 4 teeth M_2_, M_1_, PM_2_, PM_1_ (mandibular left side) in Demirjian’s 1976 method

| **Boys** | | | | | | | | | |
| --- | --- | --- | --- | --- | --- | --- | --- | --- | --- |
|  | **Stage** | | | | | | | | |
| **Tooth** | 0 | A | B | C | D | E | F | G | H |
| **M_2_** | 0.0 | 3.2 | 6.2 | 9.9 | 14.4 | 18.4 | 20.7 | 21.9 | 23.3 |
| **M_1_** |  |  |  | 0.0 | 8.0 | 12.6 | 16.9 | 21.8 | 27.4 |
| **PM_2_** | 0.0 | 3.1 | 5.6 | 9.5 | 13.7 | 17.4 | 20.1 | 21.4 | 22.5 |
| **PM_1_** |  | 0.0 | 5.9 | 10.7 | 15.7 | 20.7 | 23.8 | 25.4 | 26.8 |
| **Girls** | | | | | | | | | |
|  | **Stage** |  |  |  |  |  |  |  |  |
| **Tooth** | 0 | A | B | C | D | E | F | G | H |
| **M_2_** | 0.0 | 3.6 | 6.1 | 9.9 | 15.3 | 19.2 | 21.7 | 23.0 | 24.2 |
| **M_1_** |  |  |  | 0.0 | 5.4 | 9.8 | 14.3 | 20.1 | 25.9 |
| **PM_2_** | 0.0 | 3.7 | 5.8 | 9.8 | 14.7 | 18.1 | 20.8 | 22.3 | 23.3 |
| **PM_1_** |  | 0.0 | 4.6 | 9.2 | 15.1 | 20.2 | 23.3 | 25.1 | 26.6 |

**Source:** Demirjian A, Goldstein H (1976) New systems for dental maturity based on seven and four teeth. Ann Hum Biol 3: 411-421.
